# Unmasking the Risk of Vaping-Associated Pneumothorax in Rib Cartilage Rhinoplasty

**DOI:** 10.1055/a-2819-3130

**Published:** 2026-06-26

**Authors:** Beta S. Nata'atmadja, Pascalis Fabian, Loelita M. Lumintang

**Affiliations:** 1 Department of Plastic Reconstructive and Aesthetic Surgery, Faculty of Medicine, Airlangga University, Surabaya, Indonesia; 2 General Practitioner, Faculty of Medicine, Airlangga University, Surabaya, Indonesia; 3 Department of Reconstructive and Aesthetic Plastic Surgery, Sanjiwani General Hospital, Faculty of Medicine, Universitas Warmadewa, Denpasar, Indonesia

**Keywords:** rib rhinoplasty, pneumothorax, Iatrogenic, e-cigarettes, vaping

## Abstract

Rib cartilage grafting is a popular procedure for rhinoplasty due to its advantages of zero foreign material involvement and minimal to near zero risk of skin perforation. However, there are some potential risks associated with this procedure, including warping and pneumothorax.

Even though multiple studies have stated that the incidence rate of pneumothorax in rib graft harvest is very low, we cannot neglect its reality and probability of it happening. In this article, we highlight two rare cases of pneumothorax in rib cartilage graft rhinoplasty. The first case involved a pneumothorax that occurred during rib cartilage harvest in a 37-year-old woman. The second case involved a spontaneous pneumothorax that occurred immediately after rib cartilage rhinoplasty surgery in a 46-year-old woman.

Both pneumothoraces were managed successfully with conservative observation and chest tube placement, respectively. The patients made a full recovery and were able to return to their normal activities. One of the potential risks of pneumothorax is the use of e-cigarettes or vaping. Vaping-associated spontaneous pneumothorax has been reported in only a handful of studies. These cases highlight the importance of being aware of the potential risks of rib cartilage graft rhinoplasty and taking steps to minimize and manage them.

Using rib cartilage graft over alloplastic materials for rhinoplasty has become increasingly popular. The use of autologous cartilage offers excellent tissue fusion and reduces the chances of skin perforation without the use of foreign material. 1 Rib cartilage offers an abundant amount of material for grafting, compared with septal or auricular donors. This procedure offers stability and predictability of aesthetic and functional outcomes. 2 However, there is no perfect procedure yet as it still lurks with risks such as pneumothorax and warping. 3 This communication article highlights two rare cases of pneumothorax in rib cartilage rhinoplasty and the potential risk of vaping-associated pneumothorax.

In the first case, a 37-year-old woman with history of polydioxanone thread rhinoplasty underwent a full rib cartilage rhinoplasty using her right seventh rib. The cartilage graft was harvested using a no. 15 surgical blade and the pleura was protected using a curved periosteal elevator. During the harvest, a pleural leak was detected through bubble formation during a Valsalva maneuver. Immediate surgical treatment was done by maintaining negative pressure of −20 cm H _2_ O in the pleural area using a 20 FR nasogastric tube and suction until the closure was completed in a watertight fashion. As part of the postoperative care, thoracic surgeon consultation and surveillance thoracic X-rays were provided as shown in Fig. 1 . Since her pneumothorax did not worsen, the patient was discharged on the third surgery day with instructions on how to spot respiratory trouble.

**Fig. 1:**
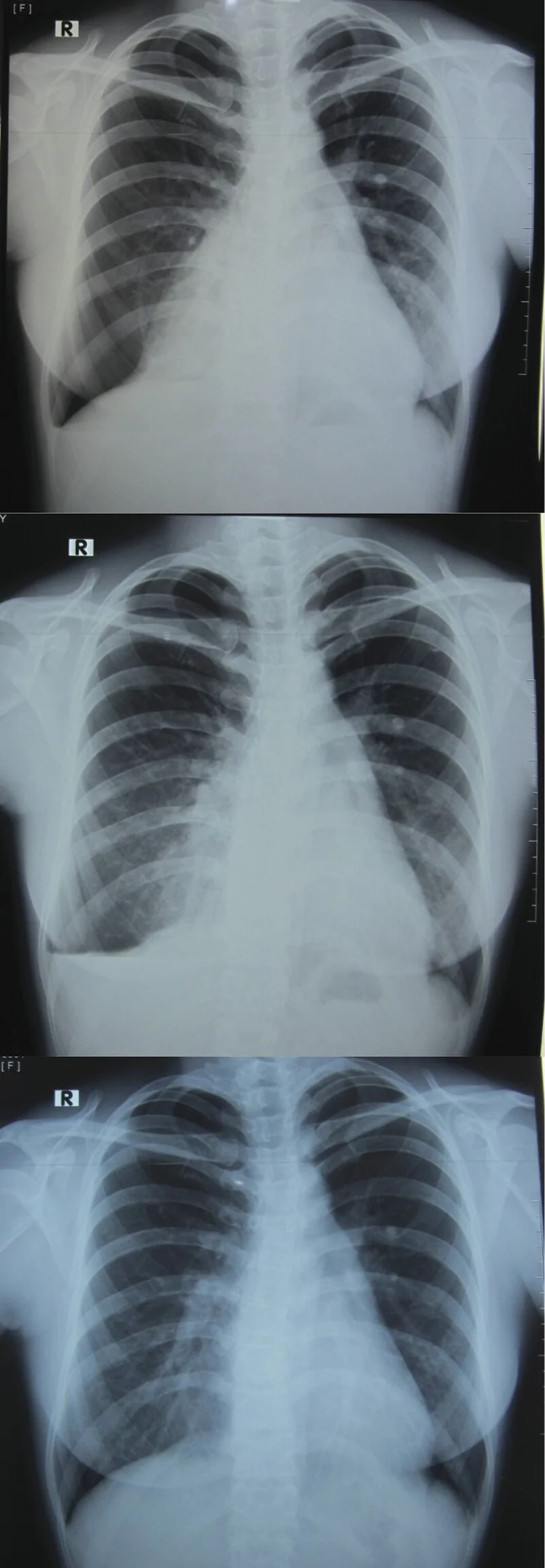
Chest X-ray sequence 1-day, 1-week, and 1-month postpneumothorax.

For the second case, a 46-year-old woman with nose siliconoma had undergone four previous silicone implant rhinoplasty and was planned for revision surgery due to implant malposition and long nose problem. The patient had a history of using e-cigarettes for around 5 years, regularly vaping around 2 times per day. The technique for graft harvesting was the same as the first case. The rib cartilage was harvested from her sixth right rib without any immediate pleural tears, as confirmed by two intraoperative Valsalva maneuvers. But postoperatively, the patient experienced a spontaneous pneumothorax, as shown by a sudden cough, breathlessness, and a decrease in oxygen saturation to 90 to 93%. Lung reinflation occurred quickly when the thoracic surgeon quickly introduced a water-sealed drainage (WSD) tube. After 3 days of WSD maintenance, the patient was given breathing exercises and released from the hospital. Later outpatient visit showed excellent recovery with no recurrence of pneumothorax, as shown in Fig. 2 .

**Fig. 2:**
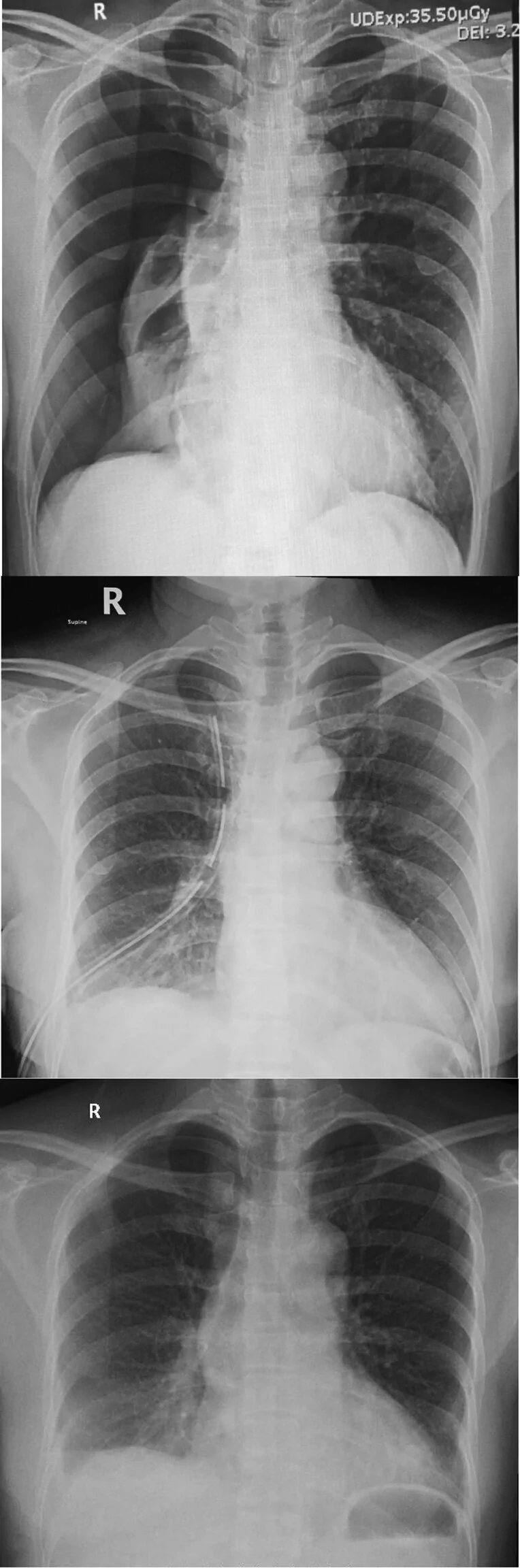
Chest X-ray sequence postoperation, post-WSD insertion, and post-WSD removal. WSD, water-sealed drainage.

These cases highlight the importance of recognizing and managing pneumothorax, a rare but serious complication in rib cartilage rhinoplasty. Surgeons must quickly diagnose this condition and employ appropriate intraoperative and postoperative measures. The use of negative pressure and careful monitoring are crucial steps in ensuring patient safety.

Pneumothorax present in both of these patients are of different cause. The first case is an example of an iatrogenic pneumothorax, whereas the second case is a spontaneous pneumothorax. Potential complications after surgery are mostly determined by the patient's medical history in addition to the technical factors. Given that the second patient had previously vaped, questions concerning the connection between e-cigarette usage and spontaneous pneumothorax are raised. While the direct causality between vaping and pneumothorax requires further investigation, existing studies suggest that vaping can lead to respiratory issues such as increased airway resistance, susceptibility to infections, and pulmonary toxicity. 4 5 6 The case of spontaneous pneumothorax in a patient with a history of vaping adds to the growing body of evidence that highlights the risks associated with e-cigarette use.

Vaping is perceived as a safer alternative to smoking, therefore increasing its popularity. However, it is essential to understand its medical effects fully. Some studies indicate that vaping can cause withdrawal symptoms, respiratory irritation, and inflammation, similar to those caused by tobacco cigarettes. 7 8 9 Some e-cigarettes even produce similar levels of nicotine compared with tobacco cigarettes. 10 These adverse effects should be considered among health care providers when evaluating patients with a history of e-cigarette use.

There are not many studies on the relation between vaping and spontaneous pneumothorax, however, similar cases have been reported as shown in five previous case reports and series that have been published. 11 12 13 14 15 The details of the studies are collected in Table 1 . However, seeing the risks and incidence of vaping-associated pneumothorax are rising, it should be further studied to properly assess this relation.

**Table 1:** Vaping-associated spontaneous pneumothorax cases

No.	Title	Age	Gender	Past history	Vaping	PSP/SSP	Coughing	Treatment
1	A case report of secondary spontaneous pneumothorax induced by Vape 11	35	M	Previous CT-scan: GGO in both lungs	48 mo	PSP	Yes	Chest tube
2	Vaping associated spontaneous pneumothorax—a case series of an enigmatic entity! 12	19	M	None	3 h before	PSP	No	Conservative
3		20	F	None	2 d before	PSP	Yes	Chest tube
4		18	M	None	2 mo	PSP	No	Chest tube
5		35	M	Spontaneous pneumothorax 8 y prior Previous CT scan: blebs in the upper lobe	3 h before	SSP	Yes	Chest tube
6	Reporting of pneumothorax in association with vaping devices and electronic cigarettes 13	34	M	Rib fracture 3 y prior smoker for 10 y, stopped 3 y prior	36 mo	PSP	No	Chest tube
7	Recurrent spontaneous pneumothorax in a 15-year-old female associated with electronic cigarettes 14	15	F	Recurrent spontaneous pneumothorax	Daily use	SSP	No	Chest tube
8	Recurrent spontaneous pneumothoraces and vaping in an 18-year-old man: a case report and review of the literature 15	18	M	None	18 mo	PSP	No	Chest tube

Abbreviations: CT, computed tomography; F, female; GGO, ground-glass opacity; M, male.

In conclusion, while rib cartilage rhinoplasty offers significant benefits, it is not without risks. Even though they are rare, the risk of pneumothorax still lurks and needs to be highly considered. Late diagnosis and management of pneumothorax could be fatal for the patient. In order to give patients the best treatment possible, surgeons need to stay alert and notice these risks. Further studies are necessary to assess the relation between vaping and spontaneous pneumothorax.
